# Supplementary material for: A message of the majority with scientific evidence encourages young people to show their prosocial nature in COVID-19 vaccination
Source: Sci Rep. 2021 Dec 1;11:23261. doi: 10.1038/s41598-021-02230-1 (PMC8636594; doi:10.1038/s41598-021-02230-1)
Supplement: Supplementary file 1 — Supplementary Information. [file 41598_2021_2230_MOESM1_ESM.pdf]

Supplementary Information for

A message of the majority with scientific evidence encourages young people to show their prosocial nature in COVID-19 vaccination

authors

Toshiko Tanaka[1], Tsuyoshi Nihonsugi[2], Fumio Ohtake[3] & Masahiko Haruno*[1,4]

affiliations

1.Center for Information and Neural Networks (CiNet), National Institute of Information and Communications Technology (NICT), Suita, Japan
2.Faculty of Economics, Osaka University of Economics, Osaka, Japan
3.Graduate School of Economics, Osaka University, Toyonaka, Japan
4.Grauduate School of Frontier Biosciences, Osaka University, Suita, Japan

Contact information
Correspondance should be addressed to Dr. Masahiko Haruno, Center for Information and Neural Networks, National Institute of Information and Communications Technology, Suita,Osaka 565-0871, Japan. E-mail: mharuno@nict.go.jp.

Figure S1. Subjective evaluation of messages.

The percentage of people who scored in each of the seven subjective ratings (responsibleness, empathy, peer pressure, repulsed, uncomfortable, stimulating, and memorable) by gender and age are shown. Feelings of responsibility were lower among men in their 10-20s in response to nudge 2 ($p = 4.9 \times 10^{-3}$). No significantly greater senses of repulsion or discomfort occurred in response to nudge 8. Significant differences compared with control (nudge 1) are indicated by the asterisks (*). The p-values of the black asterisks for feeling responsible are, from left (male) to right (female), 4.9×10^{-3}, 0.043, 7.6×10^{-3}, and 0.031, and those of the blue asterisks are 0.012, 0.024, 0.011, and 0.021; for empathy, they are 0.038, 0.035, 0.027, and 0.013 and 0.041 and 0.022, respectively; for peer pressure, they are 0.021 and 0.019 and 9.4×10^{-3}, 0.010, and 0.044, respectively; for repulsion, they are 0.031 and 0.036, respectively; for discomfort, they are 0.047 and 0.030, 0.049, and 0.028, respectively; for stimulating, they are 0.036, 0.033, and 7.1×10^{-3} and 7.4×10^{-3}, 0.011, 0.037, and 0.022, respectively; and for feeling memorable, they are 0.019, 0.045, and 0.032.

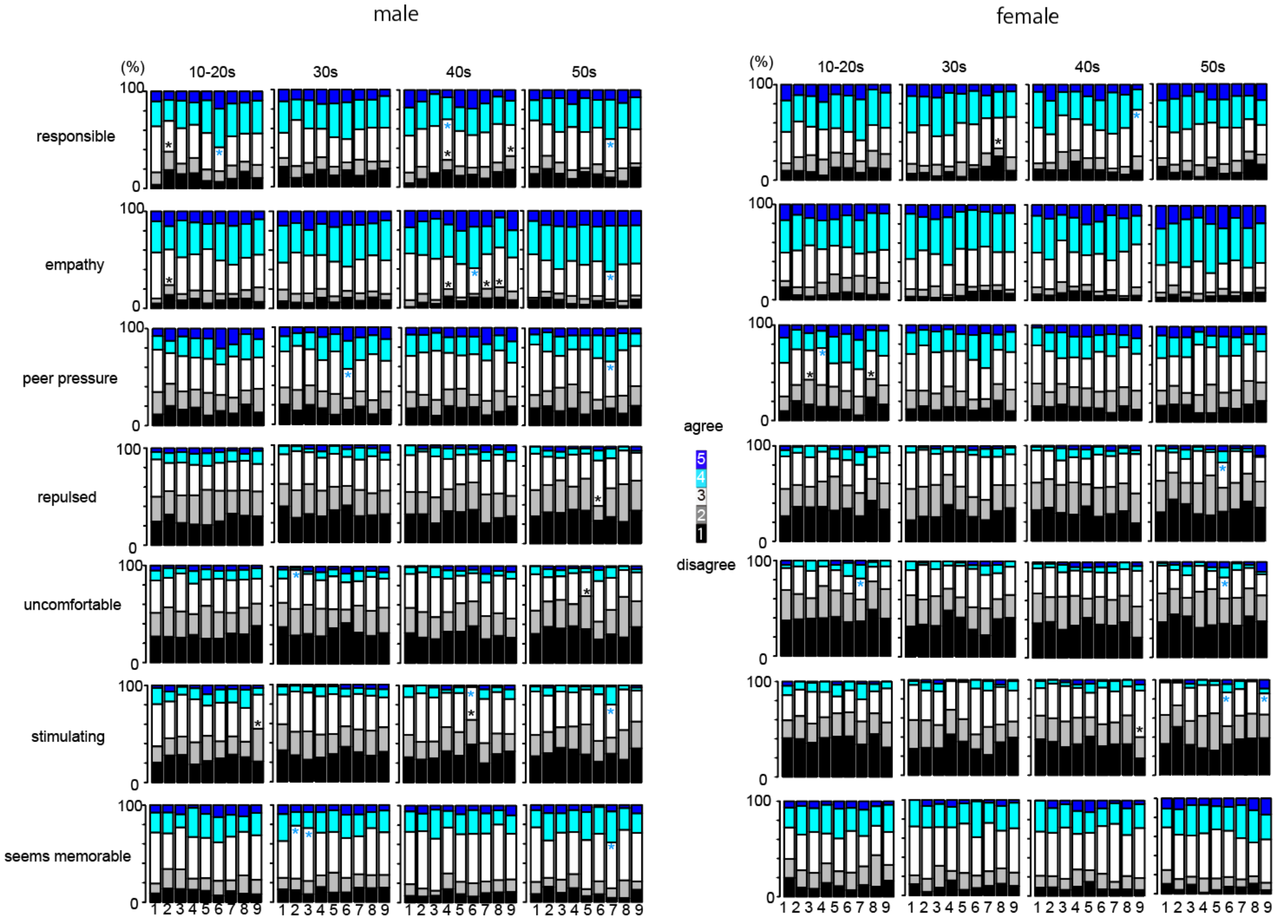

Table S1. Detailed list of the personality tests used in this study.

Abbreviation	subscore abbreviation (item number)	Questionnaire name	references
Big5		Big Five personality traits	1,2
Extraversion	Big5_E (12)		
Agreeableness	Big5_A (12)		
Consciousness	Big5_C (12)		
Neuroticism	Big5_N (12)		
Openness	Big5_O (12)		
IRI		Interpersonal Reactivity Index	3,4
Fantasy	IRI_F (7)		
Perspective taking	IRI_PT (7)		
Empathic Concern	IRI_EC (7)		
Personal Distress	IRI_PD (7)		
MVS	(20)	Machiavellianism Scale	5,6
PSS	(10)	Perceived Subjective Stress	7,8
RA	(10)	Risk Aversion	9,10
RSS	(10)	Rosenberg Self - Esteem scale	11,12
SES	(1)	Socio Economic Status	13,14
SVO		Social Value Orientation	15,16
Prosocial	SVO_P (8)		
indvidualist	SVO_I (8)		
Competitor	SVO_C (8)		
STAI		State - Trait Anxiety Inventory	17,18
State	STAI_S (20)		
trait	STAI_T (20)		
TIM	(15)	Time discounting	19,20
CCP	(1)	conditional cooperation	21
TRU			
trust	TRU_GSSWVS (1)	the World Values survey/the American General Social Survey	
fairness	TRU_WVS (1)	the World Values survey	
altruism	TRU_GSS (1)	the American General Social Survey	
LAR	loss aversion (6)	lottery choice task	22
Inequity Guilt aversion			27
guilt-aversion	guilt		
inequity-aversion	inequity		
ES		Empathizing–Systemizing theory	23
Systemizing	ES_S (20)		
Empathizing	ES_E (20)		
SHS	(4)	Tendency to give socially desirable answers	24,25
IQ	(9)	Raven Advanced Progressive Matrices test #11,24,28,36,43,48,49,53,55	26

Table S2. Subjective evaluation of the messages.

		all									male									female								
**items**	nudge	total	disagree (12)		nutral (3)		agree (45)		F/chi_sq	p_value	total	disagree (12)		nutral (3)		agree (45)		F/chi_sq	p_value	total	disagree (12)		nutral (3)		agree (45)		F/chi_sq	p_value
		n	n	%	n	%	n	%			n	n	%	n	%	n	%			n	n	%	n	%	n	%		
		6232									3409									2823								
	total		1285	20.6	2180	35.0	2627	42.2				806	23.6	1216	35.7	1387	40.7				619	21.9	964	34.1	1240	43.9		
	1	705	140	19.9	259	36.7	306	43.4	29.517	0.0207	382	80	20.9	149	39.0	153	40.1	30.59	0.0152	323	60	18.6	110	34.1	153	47.4	19.29	0.254
	2	695	162	23.3	258	37.1	275	39.6			384	105	27.3	146	38.0	133	34.6			311	57	18.3	112	36.0	142	45.7		
	3	684	155	22.7	244	35.7	285	41.7			376	84	22.3	143	38.0	149	39.6			308	71	23.1	101	32.8	136	44.2		
	4	689	163	23.7	246	35.7	270	39.2			369	98	26.6	138	37.4	133	36.0			310	65	21.0	108	34.8	137	44.2		
**responsible**	5	711	155	21.8	247	34.7	309	43.5			388	79	20.4	136	35.1	173	44.6			323	76	23.5	111	34.4	136	42.1		
	6	699	150	21.5	220	31.5	329	47.1			381	83	21.8	116	30.4	182	47.8			318	67	21.1	104	32.7	147	46.2		
	7	695	148	21.3	224	32.2	323	46.5			387	87	22.5	126	32.6	174	45.0			308	61	19.8	98	31.8	149	48.4		
	8	677	171	25.3	240	35.5	266	39.3			367	89	24.3	136	37.1	142	38.7			310	82	26.5	104	33.5	124	40.0		
	9	687	181	26.3	242	35.2	264	38.4			375	101	26.9	126	33.6	148	39.5			312	80	25.6	116	37.2	116	37.2		
	total		918	14.7	2170	34.8	3144	50.4				511	15.0	1218	35.7	1680	49.3				407	14.4	952	33.7	1464	51.9		
	1	705	92	13.0	260	36.9	353	50.1	10.85	0.82	382	47	12.3	158	41.4	177	46.3	24.39	0.0814	323	45	13.9	102	31.6	176	54.5	11.06	0.806
	2	695	107	15.4	252	36.3	336	48.3			384	66	17.2	146	38.0	172	44.8			311	41	13.2	106	34.1	164	52.7		
	3	684	94	13.7	251	36.7	339	49.6			376	50	13.3	136	36.2	190	50.5			308	44	14.3	115	37.3	149	48.4		
	4	689	102	14.8	236	34.3	341	49.5			369	65	17.6	130	35.2	174	47.2			310	37	11.9	106	34.2	167	53.9		
**empathy**	5	711	105	14.8	239	33.6	367	51.6			388	48	12.4	142	36.6	198	51.0			323	57	17.6	97	30.0	169	52.3		
	6	699	105	15.0	224	32.0	370	52.9			381	57	15.0	114	29.9	210	55.1			318	48	15.1	110	34.6	160	50.3		
	7	695	107	15.4	223	32.1	365	52.5			387	58	15.0	126	32.6	203	52.5			308	49	15.9	97	31.5	162	52.6		
	8	677	100	14.8	244	36.0	333	49.2			367	59	16.1	138	37.6	170	46.3			310	41	13.2	106	34.2	163	52.6		
	9	687	106	15.4	241	35.1	340	49.5			375	61	16.3	128	34.1	186	49.6			312	45	14.4	113	36.2	154	49.4		
	total		2088	33.5	2351	37.7	1793	28.8				1141	33.5	1324	38.8	944	27.7				947	33.5	1027	36.4	849	30.1		
	1	705	235	33.3	280	39.7	190	27.0	45.00	1.39x10-4	382	132	34.6	161	42.1	89	23.3	30.68	0.015	323	103	31.9	119	36.8	101	31.3	30.88	0.014
	2	695	243	35.0	281	40.4	171	24.6			384	137	35.7	157	40.9	90	23.4			311	106	34.1	124	39.9	81	26.0		
	3	684	241	35.2	254	37.1	189	27.6			376	132	35.1	148	39.4	96	25.5			308	109	35.4	106	34.4	93	30.2		
	4	689	244	35.4	251	36.4	184	26.7			369	138	37.4	128	34.7	103	27.9			310	106	34.2	123	39.7	81	26.1		
**peer pressure**	5	711	244	34.3	264	37.1	203	28.6			388	132	34.0	154	39.7	102	26.3			323	112	34.7	110	34.1	101	31.3		
	6	699	201	28.8	261	37.3	237	33.9			381	110	28.9	141	37.0	130	34.1			318	91	28.6	120	37.7	107	33.6		
	7	695	200	28.8	248	35.7	247	35.5			387	114	29.5	146	37.7	127	32.8			308	86	27.9	102	33.1	120	39.0		
	8	677	238	35.2	270	39.9	169	25.0			367	115	31.3	157	42.8	95	25.9			310	123	39.7	113	36.5	74	23.9		
	9	687	242	35.2	242	35.2	203	29.5			375	131	34.9	132	35.2	112	29.9			312	111	35.6	110	35.3	91	29.2		
	total		3569	6.3864	2005	289.1	655	94.458				1889	55.4	1134	33.3	386	11.3				1683	59.6	871	30.9	269	9.5		
	1	705	398	56.5	248.0	35.2	59	8.4	32.52	8.55x10-3	382	209	54.7	137	35.9	36	9.4	17	0.386	323	189	58.5	111	34.4	23	7.1	31.25	0.0125
	2	695	408	58.7	234.0	33.7	53	7.6			384	212	55.2	137	35.7	35	9.1			311	196	63.0	97	31.2	18	5.8		
	3	684	393	57.5	212.0	31.0	79	11.5			376	211	56.1	124	33.0	41	10.9			308	182	59.1	88	28.6	38	12.3		
	4	689	399	57.9	210.0	30.5	70	10.2			369	202	54.7	120	32.5	47	12.7			310	197	63.5	90	29.0	23	7.4		
**repulsed**	5	711	436	61.3	199.0	28.0	73	10.3			388	234	60.3	113	29.1	41	10.6			323	205	63.5	86	26.6	32	9.9		
	6	699	378	54.1	234.0	33.5	87	12.4			381	205	53.8	129	33.9	47	12.3			318	173	54.4	105	33.0	40	12.6		
	7	695	372	53.5	230.0	33.1	93	13.4			387	209	54.0	123	31.8	55	14.2			308	163	52.9	107	34.7	38	12.3		
	8	677	397	58.6	217.0	32.1	63	9.3			367	200	54.5	133	36.2	34	9.3			310	197	63.5	84	27.1	29	9.4		
	9	687	388	56.5	221.0	32.2	78	11.4			375	207	55.2	118	31.5	50	13.3			312	181	58.0	103	33.0	28	9.0		
	total		3761	60.3	1847	29.6	624	10.0				1934	56.7	1101	32.3	374	11.0				1827	64.7	746	26.4	250	8.9		
	1	705	434	61.6	208.0	29.5	63	8.9	35.21	3.7x10-3	382	217	56.8	124	32.5	41	10.7	17.9	0.33	323	217	67.2	84	26.0	22	6.8	30.26	0.0167
	2	695	422	60.7	224.0	32.2	49	7.1			384	220	57.3	133	34.6	31	8.1			311	202	65.0	91	29.3	18	5.8		
	3	684	407	59.5	209.0	30.6	68	9.9			376	214	56.9	127	33.8	35	9.3			308	193	62.7	82	26.6	33	10.7		
	4	689	421	61.1	193.0	28.0	65	9.4			369	205	55.6	117	31.7	47	12.7			310	216	69.7	76	24.5	18	5.8		
**unconfortable**	5	711	452	63.6	194.0	27.3	65	9.1			388	238	61.3	112	28.9	38	9.8			323	214	66.3	82	25.4	27	8.4		
	6	699	400	57.2	210.0	30.0	89	12.7			381	206	54.1	126	33.1	49	12.9			318	194	61.0	84	26.4	40	12.6		
	7	695	389	56.0	208.0	29.9	98	14.1			387	209	54.0	121	31.3	57	14.7			308	180	58.4	87	28.2	41	13.3		
	8	677	425	62.8	193.0	28.5	59	8.7			367	212	57.8	121	33.0	34	9.3			310	213	68.7	72	23.2	25	8.1		
	9	687	411	59.8	208.0	30.3	68	9.9			375	213	56.8	120	32.0	42	11.2			312	198	63.5	88	28.2	26	8.3		
			3403	54.605	2211	35.478	618	9.9				1732	50.8	1313	38.5	364	10.7				1671	59.2	898	31.8	254	9.0		
	1	705	385	54.6	250.0	35.5	70	9.9	24.626	0.077	382	189	49.5	149	39.0	44	11.5	17.46	0.357	323	196	60.7	101	31.3	26	8.0	26.13	0.0523
	2	695	380	54.7	255.0	36.7	60	8.6			384	183	47.7	162	42.2	39	10.2			311	197	63.3	93	29.9	21	6.8		
	3	684	358	52.3	263.0	38.5	63	9.2			376	187	49.7	155	41.2	34	9.0			308	171	55.5	108	35.1	29	9.4		
	4	689	387	56.2	234.0	34.0	58	8.4			369	191	51.8	141	38.2	37	10.0			310	196	63.2	93	30.0	21	6.8		
**stimulating**	5	711	406	57.1	236.0	33.2	69	9.7			388	203	52.3	146	37.6	39	10.1			323	203	62.8	90	27.9	30	9.3		
	6	699	374	53.5	258.0	36.9	67	9.6			381	201	52.8	144	37.8	36	9.4			318	173	54.4	114	35.8	31	9.7		
	7	695	358	51.5	239.0	34.4	98	14.1			387	183	47.3	147	38.0	57	14.7			308	175	56.8	92	29.9	41	13.3		
	8	677	376	55.5	242.0	35.7	59	8.7			367	185	50.4	144	39.2	38	10.4			310	191	61.6	98	31.6	21	6.8		
	9	687	379	55.2	234.0	34.1	74	10.8			375	210	56.0	125	33.3	40	10.7			312	169	54.2	109	34.9	34	10.9		
	total		1475	23.7	2846	45.7	1911	30.7				799	23.4	1618	47.5	992	29.1				676	23.9	1228	43.5	919	32.6		
	1	705	169	24.0	330.0	46.8	206	29.2	14.89	0.53	382	81	21.2	190	49.7	111	29.1	17.83	0.334	323	88	27.2	140	43.3	95	29.4	13.87	0.61
	2	695	155	22.3	327.0	47.1	213	30.6			384	91	23.7	185	48.2	108	28.1			311	64	20.6	142	45.7	105	33.8		
	3	684	154	22.5	329.0	48.1	201	29.4			376	83	22.1	190	50.5	103	27.4			308	71	23.1	139	45.1	98	31.8		
	4	689	185	26.9	288.0	41.8	206	29.9			369	103	27.9	164	44.4	102	27.6			310	82	26.5	124	40.0	104	33.5		
**memorable**	5	711	164	23.1	320.0	45.0	227	31.9			388	88	22.7	178	45.9	122	31.4			323	76	23.5	142	44.0	105	32.5		
	6	699	154	22.0	319.0	45.6	226	32.3			381	84	22.0	174	45.7	123	32.3			318	70	22.0	145	45.6	103	32.4		
	7	695	155	22.3	315.0	45.3	225	32.4			387	86	22.2	174	45.0	127	32.8			308	69	22.4	141	45.8	98	31.8		
	8	677	179	26.4	304.0	44.9	194	28.7			367	92	25.1	188	51.2	87	23.7			310	87	28.1	116	37.4	107	34.5		
	9	687	160	23.3	314.0	45.7	213	31.0			375	91	24.3	175	46.7	109	29.1			312	69	22.1	139	44.6	104	33.3		

References

1. Goldberg, L. R. The structure of phenotypic personality traits. *Am. Psychol.* **48**, 26–34 (1993).

2. Murakami, Y. & Murakami, C. Scale construction of a 'Big Five' personality inventory. *Japanese J. Personal.* **6**, 29–39 (1997).

3. Davis, M. H. Measuring individual differences in empathy: Evidence for a multidimensional approach. *J. Pers. Soc. Psychol.* **44**, 113–126 (1983).

4. Nomura, K., Akai, S. & Morikawa, K. Pilot Japanese Interpersonal Reactivity Index. *in Proceedings of the Annual Meeting of Japanese Psychological Association* (2015).

5. Chrisite, R. & Geis, F. L. *Studies is Machiavellianism.* (Academic Press, 1970).

6. Toshitake, N. et al. Development and Validation of a Japanese Version of the Machiavellianism Scale. *Japanese J. Personal.* **20**, 233–235 (2012).

7. Cohen, S., Kamarck, T. & Mermelstein, R. A Global Measure of Preceived Stress. *J. Health Soc. Behav.* **24**, 385–396 (1983).

8. Katsunori, S. Reliability and validity of the Japanese version of the Perceived Stress Scale. *Japanese J. Heal. Psychol.* **19**, 44–53 (2006).

9. Charles, A. H. & Laury, S. K. Risk aversion and incentive effects. *Am. Econ. Rev.* **92**, 1644–1655 (2002).

10. Ikeda, S., Ohtake, F. & Yoshiro, T. Time Discount Rates: An Analysis Based on Economic Experiments and Questionnaire Surveys. *ISER Discuss. Pap.* **638**, (2005).

11. Rosenberg, M. *Society and the adolescent self-image.* (Princeton University Press, 1965).

12. Shigeo, S. Investigation of the Japanese version of Rosenberg's Self-esteem Scale. *Bull. Tsukuba Dev. Clin. Psychol.* **12**, 65–71 (2000).

13. Krieger, N., Williams, D. R. & Moss, N. E. Measuring Social Class in US Public Health Research: Concepts, Methodologies, and Guidelines. *Annu. Rev. Public Health* **18**, 341–378 (1997).

14. Okada, N. et al. Brief rating scale of socioeconomic status for biological psychiatry research among Japanese people：A scaling based on an educational history. *Japanese J. Biol. Psychiatry*. **25**, 115–117 (2014).

15. Van Lange, P. A. M. & Visser, K. Locomotion in social dilemmas: How people adapt to cooperative, tit-for-tat, and noncooperative partners. *J. Pers. Soc. Psychol.* **77**, 762–773 (1999).

16. Mori, K. Current Issues in the Measurement of Social Value Orientation. *Kwansei Gakuin Sociol. Dep. Stud.* **120**, 33–51 (2015).

17. Spielberger, C. D. *State-Trait Anxiety Inventory: Bibliography.* (Consulting

Psychologists Press, 1989).

18. Shimizu, H. & Imae, K. Development of a Japanese version of the state-trait anxiety inventory. *Japanese J. Educ. Psychol.* **29,** 62–67 (1981).

19. Green, L., Fry, A. F. & Myerson, J. Discounting of delayed rewards: A Life-Span Comparison. *Psychol. Sci.* **5**, 33–36 (1994).

20. Hiruma, F. A research on background factors of time discount rate by questionnaire measure. *Waseda Commer. Sci.* **432**, 1–34 (2012).

21. Fischbacher, U., Gächter, S. & Fehr, E. Are people conditionally cooperative? Evidence from a public goods experiment. *Economics Letters,* **71**, 397-404 (2001).

22. Gächter, S., Johnson, E. J. & Herrmann, A, Individual-Level Loss Aversion in Riskless and Risky Choices. *IZA Discussion Paper.* No. 2961 (2007). https://ssrn.com/abstract=1010597

23. Greenberg, D. M., Warrier, V., Allison, C. & Baron-Cohen, S. Testing the Empathizing–Systemizing theory of sex differences and the Extreme Male Brain theory of autism in half a million people *PNAS*. **115**, 12152-12157 (2018).

24. Lyubomirsky, S. & Lepper, H. S. A measure of subjective happiness: Preliminary reliability and construct validation. *Social Indicators Research*. **46**, 137–155 (1999).

25. Shimai, S., Otake, K., Utsuki, N., Ikemi, A. & Lyubomirsky, S. Development of a Japanese version of the subjective happiness scale (SHS), and examination of its validity and reliability. *Japanese Journal of Public Health*. **10**, 845–853 (2004).

26. Jensen, A. R. *The g factor: The science of mental ability.* (Praeger, 1998).

27. Nihonsugi, T., Ihara, A. & Haruno, M. Selective increase of intention-based economic decisions by noninvasive brain stimulation to the dorsolateral prefrontal cortex. *J. Neurosci*. **35**, 3412-3419 (2015).

Appendix S1: The original messages (nudges) in Japanese for promoting vaccination used in the experiments.

(English translations are displayed in the main text.)

Description.

新型コロナウイルスに対してはワクチンの接種が有効だとされています。

1. control (altruism; gain framing)

あなたのワクチン接種は、病床数に余裕をもたらし、人の命を救うことにつながります。

2. scientific evidence (self-interest; gain framing)

米国の科学誌サイエンスの報告で、ワクチン接種後に急性の重いアレルギー症状を示した人は 6 万人に 1 人の割合であり、ワクチン接種で新型コロナの発症者が 20 分の1に減少することが示されています。

3. scientific evidence (self-interest; loss framing)

米国の科学誌サイエンスの報告で、ワクチン接種後に急性の重いアレルギー症状を示した人は 6 万人に 1 人の割合であり、ワクチンを接種しないと新型コロナの発症者が 20 倍に増加することが示されています。

4. scientific evidence (self-interest; gain framing) + altruism (gain framing)

米国の科学誌サイエンスの報告で、ワクチン接種後に急性の重いアレルギー症状を示した人は 6 万人に 1 人の割合であり、ワクチン接種で新型コロナの発症者が 20 分の1に減少することが示されています。

あなたのワクチン接種は、病床数に余裕をもたらし、人の命を救うことにつながります。

5. scientific evidence (self-interest; loss framing) + altruism (gain framing)

米国の科学誌サイエンスの報告で、ワクチン接種後に急性の重いアレルギー症状を示した人は 6 万人に 1 人の割合であり、ワクチンを接種しないと新型コロナの発症者が 20 倍に増加することが示されています。

あなたのワクチン接種は、病床数に余裕をもたらし、人の命を救うことにつながります。

6. scientific evidence (self-interest; gain framing) + altruism (loss framing)

米国の科学誌サイエンスの報告で、ワクチン接種後に急性の重いアレルギー症状を示した人は 6 万人に 1 人の割合であり、ワクチン接種で新型コロナの発症者が 20 分の1に減少することが示されています。

あなたがワクチン接種を行わないと、病床数が不足し、人の命の危険を招きます。

7. scientific evidence (self-interest; loss framing) + altruism (loss framing)

米国の科学誌サイエンスの報告で、ワクチン接種後に急性の重いアレルギー症状を示した人は 6 万人に 1 人の割合であり、ワクチンを接種しないと新型コロナの発症者が 20 倍に増加することが示されています。

あなたがワクチン接種を行わないと、病床数が不足し、人の命の危険を招きます。

8. scientific evidence (self-interest; gain framing) + majority

米国の科学誌サイエンスの報告で、ワクチン接種後に急性の重いアレルギー症状を示した人は 6 万人に 1 人の割合であり、ワクチン接種で新型コロナの発症者が 20 分の1に減少することが示されています。

世界的な調査会社であるイプソス社の調査によれば、およそ7割の人がワクチン接種に同意するとのことです。

9. scientific evidence (self-interest; loss framing) + majority

米国の科学誌サイエンスの報告で、ワクチン接種後に急性の重いアレルギー症状を示した人は 6 万人に 1 人の割合であり、ワクチンを接種しないと新型コロナの発症者が 20 倍に増加することが示されています。

世界的な調査会社であるイプソス社の調査によれば、およそ7割の人がワクチン接種に同意するとのことです。
